# Jiegeng Decoction Potentiates the Anticancer Efficacy of Paclitaxel *in vivo* and *in vitro*

**DOI:** 10.3389/fphar.2022.827520

**Published:** 2022-02-25

**Authors:** Haifang Chen, Guofeng Li, Ye Liu, Yifan Lang, Wuliang Yang, Wugang Zhang, Xinli Liang

**Affiliations:** ^1^ Jiangxi University of Chinese Medicine, Nanchang, China; ^2^ Key Laboratory of Modern Preparation of Traditional Chinese Medicine, Ministry of Education, Jiangxi University of Chinese Medicine, Nanchang, China

**Keywords:** paclitaxel, jiegeng, gancao, MDR, anti-cancer, meridian guide effect

## Abstract

Paclitaxel (PTX) has been the first-line treatment for lung cancer; however, its clinical use is limited due to multidrug resistance (MDR) and adverse effects. Thus, there is an urgent need to explore agents that can enhance the anticancer efficacy of PTX by reducing drug resistance and adverse reactions. Jiegeng decoction (JG) was used as the meridian guide drug and adjuvant drug in treatment of lung cancer. However, the mechanism of adjuvant effect was unclear. The aim of this study was to determine whether JG could potentiate the anticancer effect of PTX. Tissue distribution of PTX was detected using HPLC-MS/MS. The anti-lung cancer effect of the combination of PTX and JG in Lewis lung cancer C57BL/6J mice was evaluated based on the body weight and tumor-inhibition rate. PTX concentration in tumors was determined using HPLC-MS and *in vivo* imaging. Biochemical indices were detected using biochemical analyzer and ELISA. The anticancer mechanism of the PTX-JG combination in A549/PTX cells was elucidated based on cell proliferation, annexin V-FITC apoptosis assay, and western blotting. Tissue distribution analysis showed that the distribution of PTX increased in the lungs, liver, and heart upon administering the combination of PTX and JG. JG remarkably enhanced the anticancer effect of PTX by increasing the red blood cell and platelet counts; increasing hemoglobin, interleukin (IL)-2, and tumor necrosis factor-α levels; increasing CD4+T cells and the CD4+/CD8+ ratio; and decreasing IL-10 levels. JG administration led to the increased distribution of PTX at the tumor lesion sites and also potentiated the anticancer effect of PTX by inhibiting tumor cell proliferation and promoting apoptosis. Moreover, JG reversed PTX resistance by inhibiting the expression of lung resistance-related proteins, multiresistance protein 1, P-glycoprotein, and breast cancer-resistant protein. Furthermore, the combination of JG and PTX decreased alanine aminotransferase and aspartate aminotransferase levels and did not affect creatine kinase-MB levels. Therefore, our discovery suggests that JG increased the anticancer effect of PTX by downregulating the MDR-related protein and demonstrated a synergistic enhancement of immunity. Thus, the combination of PTX with JG shows potential in the management of lung cancer owing to its synergistic and detoxifying effects.

## Introduction

Globally, lung cancer remains the leading cause of cancer deaths and has a high incidence (Bade and Cruz, 2020; Yang et al., 2020). Owing to multidrug resistance (MDR) and adverse effects, most drugs used to treat lung cancer have limited clinical efficacy. Paclitaxel (PTX) has been used as the first-line treatment for lung cancer for several years; however, it is associated with the problems stated above (Koyanagi et al., 2021). Several derivatives of PTX and different drug dosages have been tested, which have resulted in minimal side effects due to an increase in drug concentration in the target area and a decrease in nonspecific distribution (Li et al., 2021; Urbanik et al., 2021). However, these derivatives are far from becoming novel drugs.

The main cause of MDR during chemotherapy is closely related to MDR-related transporters, which mainly include P-glycoprotein (P-gp/ABCB1/MDR1), breast cancer-resistant protein (BCRP/ABCG2), multiresistance protein 1 (MRP1/ABCC1), and lung resistance-related protein (MVP/LRP) (Mohammad et al., 2018). These efflux proteins are widely distributed in the small intestine, liver, kidneys, and other tissues, and are highly expressed especially in tumor sites. Thus, the high expression of MDR protein reduces the intracellular accumulation of drugs in the target sites and leads to chemo-resistance. Thus, downregulation of the MDR protein in tumor tissues or lesions is an effective approach to reduce the efflux of chemotherapeutic drugs and prevent drug resistance. Considering the long-drawn process of the development of efficacious and novel anticancer drugs with minimal side effects, a feasible method to solve the existing problem of drug resistance during chemotherapy in a clinical setting is by the use of combination therapy (Shen and Wen, 2018; Wang et al., 2018). Currently, several research groups are focusing on developing downregulators of drug-resistant proteins, which could sensitize chemotherapeutic drugs when used for cancer treatment (Chang et al., 2020; Eid et al., 2020).

Meridian guide drug is a special kind of medicine in traditional Chinese medicine and is considered to guide the power of the whole prescription to a certain lesion site or viscera. The guide effect is very similar to that of targeting agents. The literature shows that the combination of meridian guide drugs with certain other drugs can promote the absorption of the combined drugs (Wu et al., 2018; Liang et al., 2020). The mechanism of meridian guide action was related to the regulation of efflux proteins (Leng et al., 2011; Liang et al., 2016; Wu et al., 2016; Wu et al., 2021). Therefore, in view of the regulation of the meridian guiding drug on MDR-related protein, combining chemotherapy drugs with the meridian guide drugs may be an effective approach to reduce the drug resistance of chemotherapeutic agents.

Platycodonis Radix (Jiegeng), the dried roots of *Platycodon grandiflorum* (Jacq.) A. DC., Glycyrrhizae Radix Et Rhizoma (Gancao), the dried roots of *Glycyrrhiza uralensis* Fisch., *Glycyrrhiza inflata* Bat., and *Glycyrrhiza glabra* L*.* have been used as both medicine and food since ancient times. Clinically, Jiegeng and Gancao are often combined and used as a formulation known as Jiegeng decoction (JG) to clear lung qi, and relieve throat pain, wind, heat, cough, and other symptoms. In addition, JG serves as a meridian guide drug and is often combined with other traditional Chinese medicine. However, the meridian guide effect of JG remains unclear. Some studies have reported that Jiegeng and Gancao can improve the absorption or distribution of compatible drugs in tissues. Jiegeng can increase the distribution of other drugs in the lungs and enhance the anticancer effect of cisplatin (Li et al., 2018; Li et al., 2019). Gancao increased the systemic levels of the active components from Radix Paeoniae Alba (Xu et al., 2013). Moreover, JG has been clinically used as an adjuvant drug after chemotherapy for lung cancer (Shan, 1999); however, to the best of our knowledge, its mechanism as an adjuvant has not been reported.

In view of the meridian guide effect and clinical application of JG as an adjuvant in the treatment of cancer, and considering the possible concurrent use of JG and PTX to overcome the limitations of PTX, we speculated that JG could improve PTX levels in the lesion site when administered concurrently. Accordingly, we determined the potentiating effect of JG on the anticancer efficacy of PTX *in vivo* using a mouse model of Lewis lung cancer and *in vitro* using A549/PTX cells, and further clarified the underlying sensitization mechanisms of JG.

## Materials and Methods

### Chemicals and Reagents

MS grade acetonitrile, methanol, and formic acid were purchased from Tedia (Tedia Company Inc., Fairfield, United States). Water was purified using a Milli-Q water purification system (Millipore, Bedford, United States). PTX and docetaxel [internal standards (ISs)] were obtained from Chengdu Must Bio-Technology Co. Ltd. PTX injection was purchased from Hainan Quanxing Pharmaceutical Co. Ltd. Cy5.5-PTX (Cat. No. QY-C-Z021) was purchased from Xi’an Qiyue Biology Co. Ltd. Jiegeng (Jilin) and Gancao (Neimenggu) were purchased from Huangqinren drug store and stored in the Key Laboratory of Modern Chinese Medicine Preparation. Voucher specimens of Jiegeng and Gancao were numbered JG-20170301 and GC-20170302, respectively. All other reagents were of analytical grade.

### Animals Experiments and Cell Culture

C57BL/6J mice (18–22 g) and BALB/C nude mice were purchased from Hunan SJA Laboratory Animal Co., LTD (Hunan, China). All animals were kept in the laboratory of a small animal barrier environment at 24 ± 2°C and subjected to a 12 h/12 h light/dark cycle and were provided access to standard laboratory food and water *ad libitum* throughout the experiment. Experiments were commenced after 6 days of adaptation. All animal experiments were approved by the Animal Ethics Committee of Jiangxi University of Chinese Medicine (2017-004). Lewis lung cancer and A549/PTX cancer cell lines were obtained from the Jiangsu KeyGEN BioTech Corp., Ltd (Nanjing, China). A549/PTX cells were maintained in RPMI-1640 medium containing 10% FBS. Lewis lung cancer cells were cultured in a high-glucose DMEM medium containing 10% FBS. Cells were cultured at 37°C in an atmosphere of 5% CO_2_.

### Preparation of Jiegeng Decoction

**FIGURE 1:**
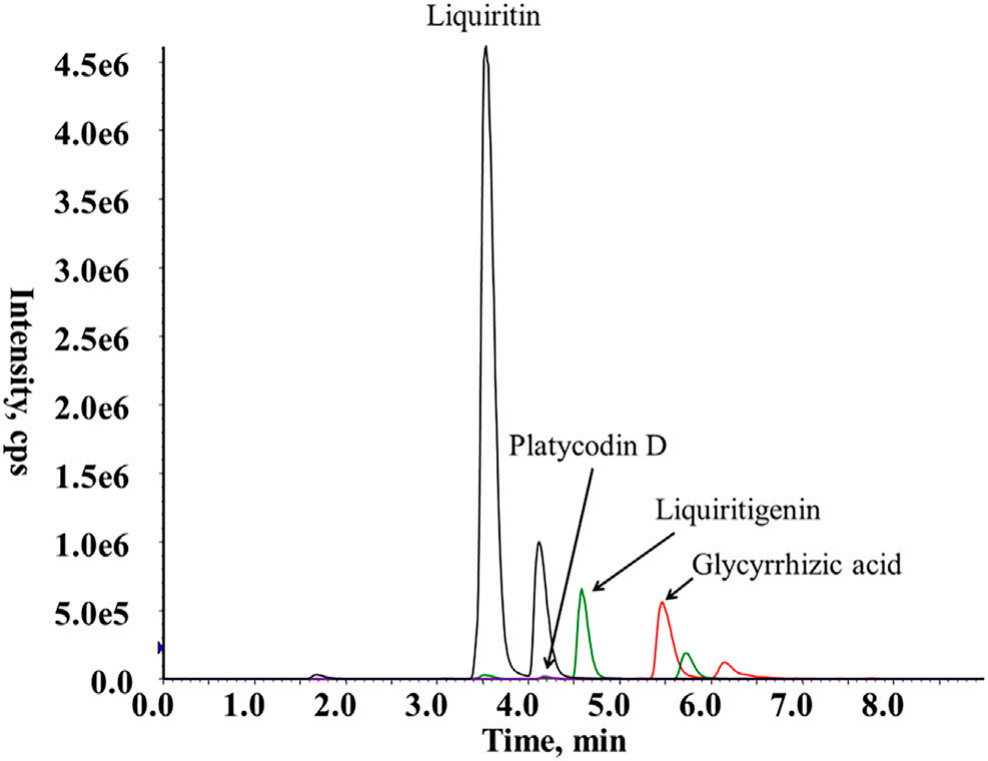
MRM chromatograms of JG.

Jiegeng and Gancao pieces were accurately weighed in a 1:1 ratio and soaked overnight in 10 times the volume of distilled water. Then, the mixture was boiled for 1 h twice and filtered. The filtrates were combined, concentrated to an appropriate volume, and frozen. After freeze-drying, JG was obtained as a powder at a yield of 49.45%. Using HPLC-MS, the contents of platycodin D, glycyrrhizic acid, liquiritin, and liquiritin were determined to be 0.136, 2.62, 0.22, and 1.87%, respectively. The mass spectra of platycodin D, glycyrrhizic acid, liquiritin, and liquiritinin from JG are shown in Figure 1.

### LC-MS

A UPLC system (Shimadzu, Japan) connected to a Triple Quad 5500MS system (AB SCIEX, Foster City, CA) with electrospray ionization (ESI) was used for UPLC-MS/MS. The UPLC system consisted of a DGU-20A_5R_ degassing unit, two LC-30AD pumps, a SIL-30AC auto-sampler, and a CTO 30A column oven. All samples were analyzed in an Agilent Zorbax extend C18 column (3.5 μm, 2.1 × 100 mm). The solvent flow rate was 0.3 ml/min and the column temperature was set at 25°C. The mobile phase was composed of acetonitrile and 0.1% formic acid in water. The elution conditions were as follows: 0–3 min, 45%–95% acetonitrile; 3–3.1 min, 90%–45% acetonitrile; and 3.1–6 min, 45% acetonitrile. The samples were analyzed in positive mode using ESI and quantified using multiple reactions monitoring (MRM). The ion pairs used for quantitative analysis were 854.5→286.1 (PTX) and 830.5→549.2 (ISs) in positive mode. The optimized MS parameters were as follows: ion spray voltage, 5.5 kV; source temperature (TEM), 550°C; curtain gas (CUR), 35 psi; ion source gas 1 (GSI) 50 psi; ion source gas 2 (GS2) 50 psi; collision gas (CAD) 7. The collision energy and declustering potential were set at 20 eV and 109 V, respectively, for PXT, and at 35 eV and 157 V, respectively, for the ISs.

### Preparation of Paclitaxel and Internal Standards, and Quality Control

Calibration samples were freshly prepared by adding 10 µl of the ISs and 10 µl of different concentrations of PTX solutions to 50 µl of blank tissue or 300 µl of blank tumor tissue. The ratio of peak area of PTX and ISs (y) was used to obtain the linear regression of PTX concentrations (x) in various tissues using partial minimum weighted multiplication. The regression equation, correlation coefficient, and linear range of standard curve of PTX in various tissues were obtained. Similarly, QC samples were prepared using blank tissue at final concentrations of low, medium, and high.

### Method Validation

The specificity of the proposed method was evaluated by comparing the blank biological tissue, PTX-spiked media, and the biological media obtained after the co-administration of JG and PTX, and JG alone, to exclude any potential interferences. Precision and accuracy were assessed at high, medium, and low concentrations using five replicates for each. Three standard stock solutions of PTX were added to the blank tissue, and the intraday and interday precision for three consecutive days were determined based on the relative standard deviation (RSD%). Accuracy was calculated as the relative error (RE%) by comparing the differences between the measured and theoretical concentrations. The recovery of PTX was calculated by comparing the peak areas of the extracted samples with those of post-extracted samples at the same concentrations. The matrix effect was calculated based on the peak area ratios of post-extracted PTX samples with those of standard PTX solutions. The stability of PTX in different biological media was assessed at three levels under various storage conditions: room temperature for 8 h, three freeze (−20°C) and thaw (room temperature) cycles, and storage at −20°C for 30 days.

### Tissue Distribution of Paclitaxel and Determination of Paclitaxel in Tumor Tissues

C57BL/6J mice were injected with PTX via the tail vein at 6.3 mg/kg either alone (n = 6), or 1 h after an oral gavage of 2.6 g/kg JG (n = 6). Mice were sacrificed at 0.1667, 0.25, 0.5, 1, 2, 4, and 8 h after PTX injections. The heart, liver, spleen, and kidneys were dissected and homogenized with six times their weight of normal saline (1:6, g: ml), whereas for lung tissues, three times the weight of normal saline was used (1:3, g: ml). After centrifugation, 100 μl of the homogenates were extracted with 800 μl acetonitrile and the supernatant was used for LC–MS/MS. The main tissue-distribution parameters from noncompartmental model analysis were calculated using DAS 3.3.0 software (Mathematical Pharmacology Professional Committee of China, Shanghai, China).

The Lewis lung cancer mouse model was established and administered the test drug as described as the preparation of Lewis lung cancer C57BL/6J mice. Mice were sacrificed 8 h after the last day of injection. Tumor tissues were processed and analyzed using LC–MS/MS.

### Establishment of Lewis Lung Cancer C57BL/6J Mice and Tumor Implantation

Lewis lung cancer cells were counted and adjusted into a single cell suspension at a concentration of 5 × 10^6^ cells/mL. C57BL/6J mice were subcutaneously injected with 0.2 ml of the cell suspension under the armpit of their right forelimb to establish a Lewis lung cancer-bearing model. When the tumor diameter was about 5–8 mm, the tumor-bearing mice were randomly divided into the following 6 groups: model group (control, normal saline), PTX group [PTX injection (3.64 mg/kg)], PTX-JG (1.3 g) group [PTX (3.64 mg/kg) combined with low-dose JG (1.3 g crude drug/kg)]; PTX-JG (2.6 g) group (PTX (3.64 mg/kg) combined with medium-dose JG (2.6 g crude drug/kg), PTX-JG (5.2 g) group (PTX (3.64 mg/kg) combined with high-dose JG (5.2 g crude drug/kg); and JG (2.6 g) group [JG (2.6 g crude drug/kg)]. Mice in the normal group were given water daily by gavage. PTX injections were administered via the tail vein every other day, whereas JG was administered daily by gavage. The drug was administered continuously for 2 weeks.

### Body Weight, Tumor Inhibition Rate, and Determination of Biochemical Indices

Mice were weighed and the tumor volume was measured with vernier caliper every other day. Mice were sacrificed after 24 h following the last administration and the weight reduction percentage (%) was calculated as follows: body-weight reduction percentage (%) = (body weight before administration—body weight after tumor removal)/body weight before administration × 100%. The heart, liver, spleen, lungs, kidneys, and the tumors of mice were dissected and weighed. The organ indices and tumor-inhibition parameters were calculated using the following formulae: Organ index (mg/g) = organ weight (mg)/body weight on the day of execution (g). Tumor volume (mm^3^) = (length ×width ×width)/2. Relative tumor volume (RTV) = Tumor volume in the administration group/Tumor volume in the model group. Relative tumor growth rate = RTV in the administration group/RTV in the model group. Tumor inhibition rate (%) = (tumor weight in the model group—tumor weight in the administration group)/tumor weight in the model group × 100%. Blood was collected from the ocular fundus venous plexus into an Eppendorf (EP) tube containing EDTA-2K. White blood cells (WBCs), red blood cells (RBCs), and platelet (PLT) counts, and hemoglobin (HGB) levels in plasma were detected using an automatic hematology analyzer. Interleukin (IL)-2, IL-10, IL-6, tumor necrosis factor-α (TNF-α), and creatine kinase-MB (CK-MB) levels were determined using ELISA, based on the optical density of samples. Total protein (TP), alanine aminotransferase (ALT), aspartate aminotransferase (AST), albumin (ALB), and alkaline phosphatase (ALP) levels in serum were detected using an automatic biochemical analyzer.

### *In vivo* Imaging

The Lewis lung cancer nude mouse model was established using a similar procedure as that described for the establishment of the Lewis lung cancer model using C57BL/6J mice. Mice were divided into the following four groups: PTX, PTX-JG (1.3 g), PTX-JG (2.6 g), and PTX-JG (5.2 g). To track PTX distribution *in vivo*, mice were injected 3.1 mg/kg of Cy5.5-PTX via the tail vein. Fluorescence intensity was observed at an excitation wavelength of 684 nm and emission wavelength of 710 nm at 0, 8, and 25 h. The intensity of Cy5.5-PTX fluorescence in tumors was quantitatively analyzed using an IVIS LuminaK *in vivo* imaging system (Caliper Life Sciences, Hopkinton, MA, United States).

### Flow Cytometry of T-Lymphocyte Subsets

The spleens of mice were taken out and placed in a 6-well plate containing 1 ml of pre-cooled phosphate-buffered saline (PBS), 24 h after the last administration. After homogenizing, the cells were filtered through a 200-mesh sieve, lysed with 600 μl of 1 × red blood cell lysate on ice for 15 min in the dark. After centrifugation at 350 g for 5 min, the cells were resuspended with PBS. This step was performed twice. Next, each spleen lymphocyte suspension (100 μl) was stained with FITC anti-mouse CD4 antibody (100405, Biolegend, San Diego, United States), APC anti-mouse CD8a antibody (100711, Biolegend, San Diego, United States), or PE anti-mouse CD3 antibody (100205, Biolegend, San Diego, United States) for 25 min in the dark. After washing twice with pre-cooled PBS containing BSA, the spleen lymphocytes were analyzed using a BD FACSVerse flow cytometer (Becton Dickinson, San Jose, CA, United States). Data were processed using FlowJo X 10.0.7r2 (BD, Ashland, United States).

### Cell Viability and Cell Proliferation Assays

Briefly, 100 μl aliquots of the cell suspension were seeded into 96-well plates. After 24 h, A549/PTX cells were treated with different concentrations of JG (8, 7, 6, 5, 4, 3.5, 3, and 2.5 mg/ml). After completion of experiments, the viability of A549/PTX cells was determined using a CCK-8 assay. Briefly, 10 μl of CCK-8 solution was added to each well of the microplate and incubated for 1 h. The absorbance was detected at 450 nm using a microplate reader (TECAN, Austria). The dose-effect curve of JG was constructed with the drug concentration as abscissa and cell survival rate as ordinate. A549/PTX cells were treated with different concentrations of PTX (60, 30, 15, 5, 2, 1, 0.5, and 0.25 μg/ml) and JG (2.5 mg/ml). The proliferation inhibition curve of PTX combined with JG for A549/PTX cells was constructed and the IC_50_ values and fold of drug resistance reversal were calculated. Reversal index = IC_50_ (PTX monotherapy)/IC_50_ (PTX combined with JG).

### Annexin V-FITC Apoptosis Assay

An assay to determine apoptosis was performed using an Annexin V-FITC/PI double-staining using flow cytometry following the manufacturer’s instructions. A549/PTX cells were incubated in 6-well plates at a density of 2 × 10^5^ cells/well. After 24 h, the cells were randomly divided into the control, PTX, JG, and combination group. After 24 h of treatment with the corresponding drugs, cells in each group were washed twice with pre-cooled PBS, digested with trypsin without EDTA and centrifuged. Next, the cells were washed twice with precooled PBS and centrifuged again. Finally, 100 μl of 1 × binding buffer were added to resuspend the cells, and 5 μl annexin V-FITC and 10 μl PI stabilizing solution were added. The cell suspension was gently shaken and incubated in the dark for 15 min. The samples were analyzed using flow cytometry within 1 h. The data were analyzed using FlowJo.

### Western Blotting

Cells and tissues were lysed with RIPA lysate (containing 1 μl PMSF and 1 μl of a phosphatase inhibitor) and the protein concentration was determined using a bicinchoninic acid kit. Samples were separated using sodium dodecyl sulfate-polyacrylamide gel electrophoresis and transferred to polyvinylpyrrolidone fluoride membranes. The membranes were blocked and incubated overnight at 4°C with antibodies to P-gp, MRP1, LRP, BCRP, and β-actin. Then, the membranes were washed and incubated with the corresponding secondary horseradish peroxidase-conjugated antibodies. Protein signals were visualized using a chemiluminescence system. The gray value of protein was determined using Image J.

### Statistical Analysis

Data were analyzed using analysis of variance (ordinary one-way ANOVA) using Prism 7.0 (GraphPad Software Inc., La Jolla, CA, United States). Data are presented as mean ± standard deviation (SD). Two-sided *p* < 0.05 was considered statistically significant.

## Results

### Method Validation

The analytical method was validated for its specificity, linearity, precision, repeatability, stability, and recovery of PTX from tissue. All calibration curves showed good linear regression within the tested ranges. The RSD for interday and intraday precision was less than 12.2%, the accuracy bias was −10.26–11.69%, and the RSD for stability was less than 13.58%. The MS chromatograms of PTX are shown in Supplementary Figure S1. The precision and recovery data were showed in Table 1. All other data are illustrated in Supplementary Table S1, S2.

**TABLE 1:** Precision, accuracy and recovery of PTX in tissue using HPLC-MS/MS (Mean ± SD, n=5).

Type of matrics	Concentration (ng/mL)	Precision						Extraction Recovery (%)	Matrix effect (%)
		Found (ng/mL)	Intra-day (RSD, %)	(RE, %)	Found (ng/mL)	Inter-day (RSD, %)	(RE, %)			
Heart	50	48.51 ± 2.23	4.60	−2.98	49.57 ± 2.85	5.74	−0.86	109.45 ± 7.63	110.86 ± 8.27
	500	498.29 ± 20.55	4.12	−0.34	554.25 ± 52.67	9.50	10.85	104.40 ± 4.56	108.92 ± 5.65
	4000	3884.66 ± 222.20	5.72	−2.88	4239.00 ± 375.08	8.85	5.98	94.35 ± 4.31	111.58 ± 8.44
Liver	200	192.64 ± 12.17	6.32	−3.68	190.98 ± 16.11	8.44	−4.51	91.49 ± 11.59	125.88 ± 3.82
	2000	1985.95 ± 72.80	3.67	−0.70	1971.05 ± 97.13	4.93	−1.45	97.32 ± 2.85	117.14 ± 15.49
	16000	14805.80 ± 528.08	3.57	−7.46	15064.58 ± 739.94	4.91	−5.85	98.52 ± 3.39	107.14 ± 3.73
Spleen	50	47.72 ± 4.07	8.53	−4.55	47.31 ± 2.72	5.75	−5.38	109.45 ± 7.63	110.86 ± 8.27
	500	502.17 ± 36.81	7.33	0.43	499.66 ± 41.56	8.32	−0.07	104.40 ± 4.10	108.92 ± 5.65
	4000	3772.16 ± 350.12	9.28	−5.70	3669.91 ± 244.09	6.65	−8.25	94.35 ± 4.31	107.24 ± 7.82
Lung	100	97.57 ± 3.13	3.21	−2.43	95.66 ± 7.00	7.32	−4.34	92.92 ± 3.07	107.28 ± 4.30
	1000	897.37 ± 39.93	4.45	−10.26	997.73 ± 136.75	13.71	−0.23	91.19 ± 3.71	96.72 ± 4.04
	8000	8891.49 ± 341.55	3.84	11.14	8787.73 ± 950.98	10.82	9.85	114.00 ± 2.74	113.35 ± 13.82
Kidney	100	97.42 ± 4.05	4.15	−2.58	98.57 ± 7.12	7.22	−1.43	111.49 ± 4.63	90.97 ± 2.43
	1000	1034.75 ± 65.77	6.36	3.47	1031.84 ± 84.68	8.21	3.18	119.25 ± 6.71	86.92 ± 4.12
	8000	8289.73 ± 315.95	3.81	3.62	8334.17 ± 228.20	2.74	4.18	126.02 ± 5.66	81.51 ± 3.36
Tumor	50	55.85 ± 6.81	12.19	11.69	47.98 ± 4.49	9.36	−4.05	83.17 ± 1.25	102.09 ± 8.19
	200	217.87 ± 22.43	10.30	8.94	205.38 ± 22.27	10.84	2.69	92.33 ± 9.76	113.57 ± 3.46
	4000	4010.97 ± 271.76	6.78	0.27	4041.66 ± 395.61	9.79	1.04	95.81 ± 3.53	111.65 ± 13.09

### Tissue Distribution of Paclitaxel in the Presence of Jiegeng Decoction

**FIGURE 2:**
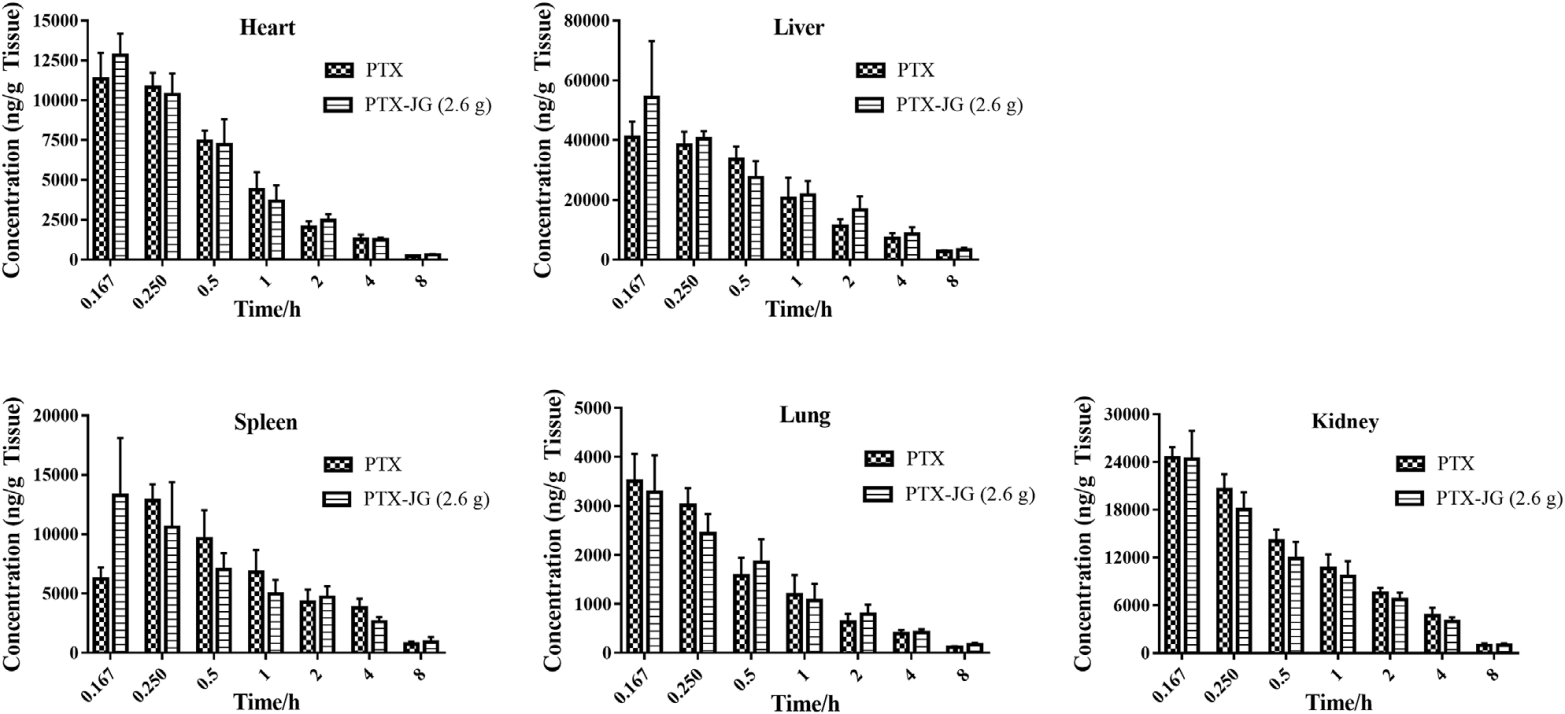
Distribution of PTX in tissue of C57BL/6J mice after the combination of JG at 0.167, 0.25, 1, 2, 4 and 8 h.

The effect of JG on the tissue distribution of PTX was assessed in the main tissues and the tissue distribution of PTX in the heart, liver, spleen, lungs, and kidneys are shown in Figure 2. The mean PTX content was the highest in the liver, followed by the kidneys, spleen, heart, and lungs. The tissue-distribution parameters of PTX in various tissues are shown in Table 2. The distribution parameters of PTX in tissue changed after the administration of JG. As shown in Figure 2, after a tail vein injection of PTX, the drug rapidly accumulated in the liver, followed by the kidneys, spleen, heart, and lungs. The AUC _(0-∞)_ of PTX in the heart, livers, and lungs increased and showed a significant increase (*p* < 0.05) after the combination. The MRT _(0-∞)_ of PTX in lungs increased and CL_z_ decreased significantly (*p* < 0.05) after administration of the combination compared with that of PTX when used alone.

**TABLE 2:** Mean pharmacokinetic parameters of PTX after the combination with JG.

Parameters	Heart		Liver		Spleen		Lung		Kidney	
	PTX	PTX–JG (2.6 g)	PTX	PTX–JG (2.6 g)	PTX	PTX–JG (2.6 g)	PTX	PTX–JG (2.6 g)	PTX	PTX–JG (2.6 g)
AUC_(0-∞)_ ( μg/L*h)	5832.9 ± 317.7	6519.4 ± 563.1^*^	32384.7 ± 4074.5	39386.5 ± 5503.1^*^	11248.9 ± 1194	11003.9 ± 1879.1	5467.2 ± 392.5	6363.1 ± 873.4^*^	17111.1 ± 1086.2	16340.5 ± 1178.8
C_max_ (μg/L)	3915.8 ± 385.6	4272.1 ± 457.0	13575.1 ± 1567.2	18226.5 ± 6159.8	4332.0 ± 466.3	4795.8 ± 1427.6	3538.5 ± 529.2	3282.46 ± 749.6	8201.2 ± 431.9	8117.7 ± 1197.2
T_1/2_ (h)	1.85 ± 0.30	1.97 ± 0.28	2.79 ± 0.76	2.39 ± 0.14	2.35 ± 0.4	2.67 ± 0.72	1.93 ± 0.34	2.87 ± 0.41^**^	1.88 ± 0.24	2.17 ± 0.34
CL_z_ (L/h/kg)	1.08 ± 0.06	0.97 ± 0.08^*^	0.20 ± 0.03	0.16 ± 0.03^*^	0.57 ± 0.06	0.58 ± 0.09	1.16 ± 0.08	1.01 ± 0.14^*^	0.37 ± 0.02	0.39 ± 0.03
MRT_(0-∞)_ (h)	1.98 ± 0.15	2.12 ± 0.21	13575.1 ± 1567.2	18226.5 ± 6159.8	3.29 ± 0.45	3.41 ± 0.65	2.33 ± 0.40	3.17 ± 0.45^**^	2.47 ± 0.19	2.7 ± 0.26

Data are presented as mean ± SD.

^*^ *p* < 0.05, ^**^ *p* < 0.01, vs. PTX alone.

AUC_(0-∞)_: area under the tissue drug concentration-time curve; CL_z_, clearance rate; MRT_(0-∞)_, mean residence time; C_max_, peak concentration; T_1/2_, half-time.

### Effect of Jiegeng Decoction on the Anti-lung Cancer Effect of Paclitaxel Using a Lewis Lung Cancer Mice Model

#### Effect of the Combination of Jiegeng Decoction and Paclitaxel on Tumor Inhibition

**FIGURE 3:**
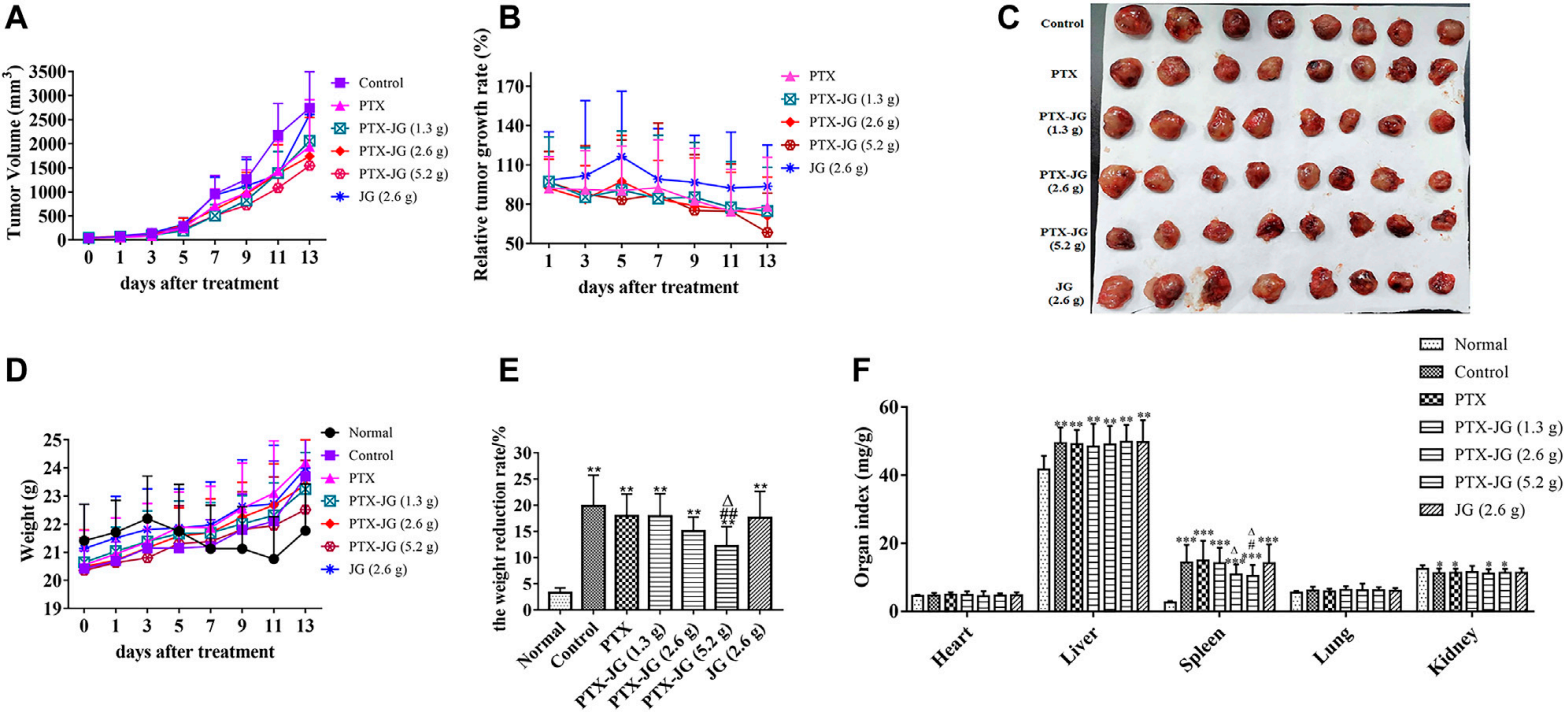
Effect of JG on the anti-lung cancer effect of PTX in Lewis lung cancer mice. **(A)**: Tumor Volume (n = 12). **(B)**: Relative tumor growth rate (n = 12). **(C)**: Tumor morphogram after the combination of PTX with JG. **(D)**: Body weight (n = 12). **(E)**: Weight reduction rate (n = 12). **(F)**: Organ indices of mice after the combination of JG and PTX (n = 15). *p < 0.05, **p < 0.01, *vs* Normal group; ^#^ *p* < 0.05, ^##^ *p* < 0.01, *vs* Control group; ^△^ *p* < 0.05, ^△△^ *p* < 0.01, *vs* PTX group.

As shown in Figure 3A and Figure 3B, tumor volume increases with days. Tumor volume and relative growth rate decreased after PTX administration, especially after PTX combined with JG (5.2 g). After the experiment finishing, the tumor tissue was stripped out and showed in Figure 3C. As shown in Table 3, tumor weight of mice in PTX group significantly decreased compared with mice in the control group (*p* < 0.01). Tumor weight of mice in combination of PTX with JG further significantly decreased compared with mice in the control group (*p* < 0.01). Table 3 also showed that tumor weight of mice in JG group was near to that of mice in control group and the antitumor activity of JG was only 14%, which could be regarded as ineffective. The antitumor effect in mice administered PTX alone was 39.99%, whereas that in mice administered the combination increased as the dose of JG was increased. The antitumor effect of high-dose JG combined with PTX was 54.57%, indicating the synergistic effect of the combination.

**TABLE 3:** Effect of combination of JG with PTX on tumor inhibition.

Group	Tumor weight	Inhibition rate (%)
Control	4.92 ± 1.61	—
PTX	2.95 ± 0.68^**^	39.99 ± 13.81
PTX–JG (1.3 g)	2.80 ± 0.44^**^	43.11 ± 8.90
PTX–JG (2.6 g)	2.62 ± 0.75^**^	46.82 ± 15.22
PTX–JG (5.2 g)	2.24 ± 0.40^**^	54.57 ± 8.19^#^
JG (2.6 g)	4.23 ± 0.55^##^	14.14 ± 11.17^#^

Data are presented as mean ± SD.

^*^ *p* < 0.05, ^**^ *p* < 0.01, vs. Control group;^#^ *p* < 0.05, ^##^ *p* < 0.01, vs. PTX group alone.

#### Effect of the combination of Jiegeng Decoction and Paclitaxel on the body weight and organ indices of mice

With the rapid increase in tumor growth in the later stage, the rate of tumor growth exceeded that of the growth of the body (Figure 3D). Therefore, to accurately evaluate the effect of drug intervention on body weight, the body weights of mice before and after intervention were recorded after tumor tissue removal. As shown in Figure 3E, the weight reduction rate in tumor-bearing mice was significantly increased (*p* < 0.01) compared with mice in the normal group, whereas it decreased after PTX treatment although the difference was not significant (*p* > 0.05). After administering the combination of JG and PTX, the weight reduction rate of mice in the PTX-JG (5.2 g) group decreased significantly compared with that of control group (*p* < 0.01).

The organ indices of mice were calculated to investigate the effect of the combination of PTX and JG, and no significant differences in the heart and lung indices were found among groups (Figure 3F). The liver index was significantly increased in tumor-bearing mice (*p* < 0.01) than in normal mice, and no differences were observed between the tumor-bearing mice. Spleen hypertrophy was observed in tumor mice in our study. Compared with mice in the normal group, the spleen index of mice in the other groups increased significantly (*p* < 0.001). Spleen hypertrophy decreased after the combination of JG and PTX, and there was a significant difference in the PTX-JG (5.2 g) group (*p* < 0.05) compared with the group that received PTX alone. The renal indices of all tumor-bearing mice were decreased compared with those in the normal group.

#### Effects of the Combination of Jiegeng Decoction and Paclitaxel on Routine Peripheral Blood

**FIGURE 4:**
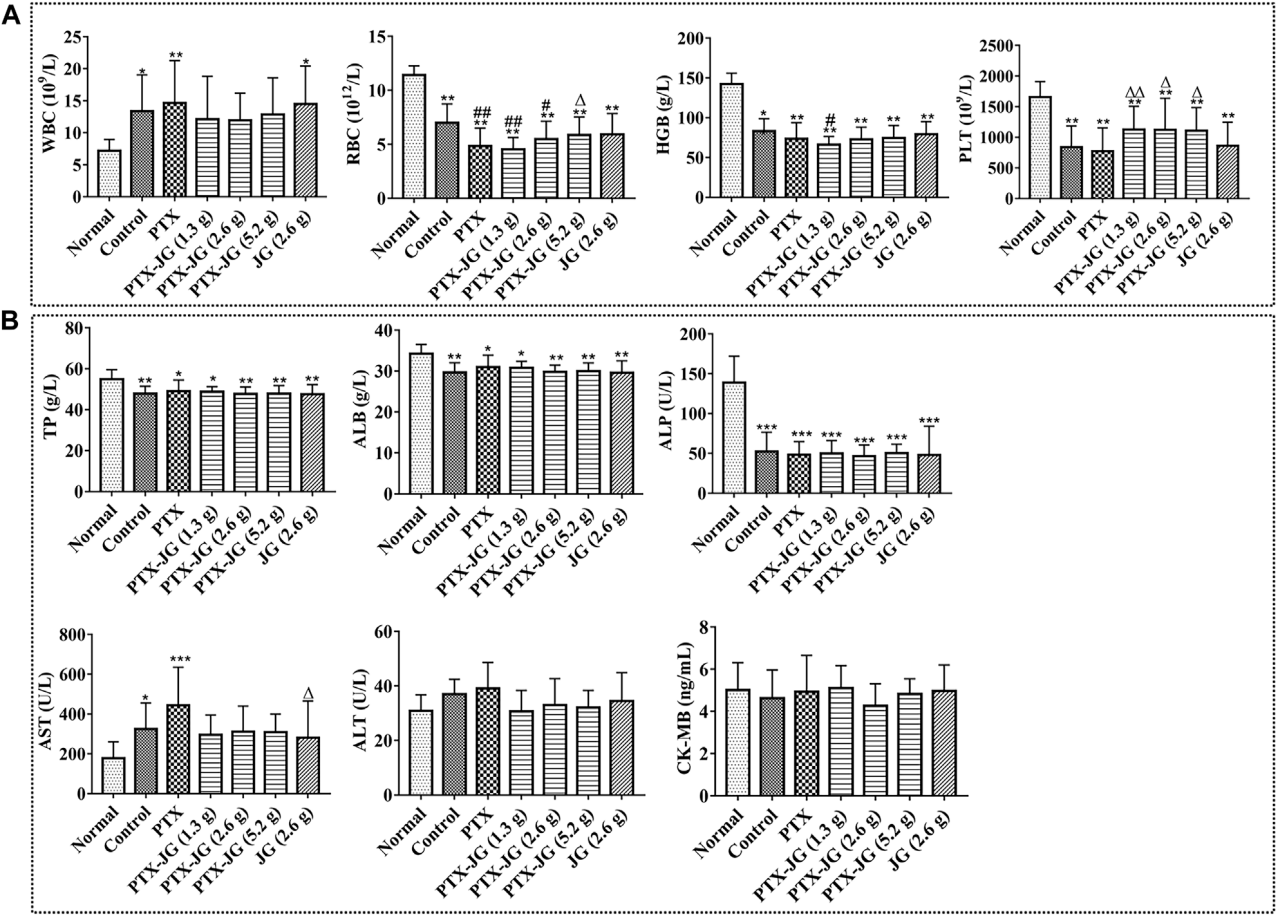
Biochemical indices after the combination of JG with PTX in the Lewis lung cancer mice. **(A)**: Routine peripheral blood (WBC, RBCs, HGB and PLTs) were detected by assay kit (n = 12). **(B)**: TP, ALB, ALP, ALT, AST and CK-MB levels were detected by assay kit (n = 10). *p < 0.05, **p < 0.01, ***p < 0.001, *vs* Normal group; ^#^ *p* < 0.05, ^##^ *p* < 0.01, *vs* Control group.^△^ *p* < 0.05, ^△△^ *p* < 0.01, *vs* PTX group.

As shown in Figure 4A, the WBC counts of tumor-bearing mice increased significantly in the control, PTX, and JG groups (*p* < 0.01, *p* < 0.05) compared with those in the normal group. WBC counts decreased after administration of the combination of JG and PTX. RBC counts in all tumor-bearing mice decreased significantly (*p* < 0.01) compared with those in the normal group. The RBC count of mice in the PTX group decreased significantly (*p* < 0.01) compared with those in the control group. After administration of the combination of JG and PTX, RBC counts increased with the increased dosages of JG. The RBC counts of mice receiving the PTX-JG (5.2 g) combination were significantly different (*p* < 0.05) compared with those receiving PTX alone. The HGB levels of all tumor-bearing mice decreased significantly (*p* < 0.01) compared with those in the normal group. HGB level increased after the combination administration of PTX with JG with the increased dosages of JG, but there was no significant difference. The PLT levels of all tumor-bearing mice decreased significantly (*p* < 0.01) compared with those in the normal group. Due to the use of PTX in the treatment process, the PLT level of mice in the PTX group further decreased compared with those in the control group; however, this change was not different. However, the combination of PTX and JG increased PLT levels and showed a significant difference compared with treatment using PTX alone (*p* < 0.01, *p* < 0.05).

#### Effects of the Combination of Jiegeng Decoction and Paclitaxel on the Liver and Heart Function of Mice

Tissue distribution studies showed that the combination of JG and PTX could increase PTX distribution in the liver. Therefore, liver function tests were performed to investigate whether increased PTX levels in the liver could aggravate PTX-induced hepatotoxicity. As shown in Figure 4B, the total protein (TP), albumin (ALB), and alkaline phosphatase (ALP) levels in tumor-bearing mice were relatively lower compared with those of mice in the normal group due to the pathological state (*p* < 0.05 or *p* < 0.01), and did not change even after treatment. AST levels of mice in the control group increased significantly compared with those of mice in the normal group. AST levels increased significantly (*p* < 0.01) after the administration of PTX. AST levels decreased after treatment with the PTX-JG combination; however, there were no significant differences between the compatibility group and the normal group or the control group. ALT levels in the control group increased compared with those in the normal group, but the difference was not significant. ALT levels in mice increased after PTX administration, but the difference was not significant compared with mice in the normal group. ALT levels decreased after administration of the JG-PTX combination, although there were no obvious significant differences compared with the normal or control groups. Furthermore, serum CK-MB levels were also determined; however, no significant differences in CK-MB levels were found among groups (Figure 4B).

#### Effects of the Combination of Jiegeng Decoction and Paclitaxel on Immunity in Mice

**FIGURE 5:**
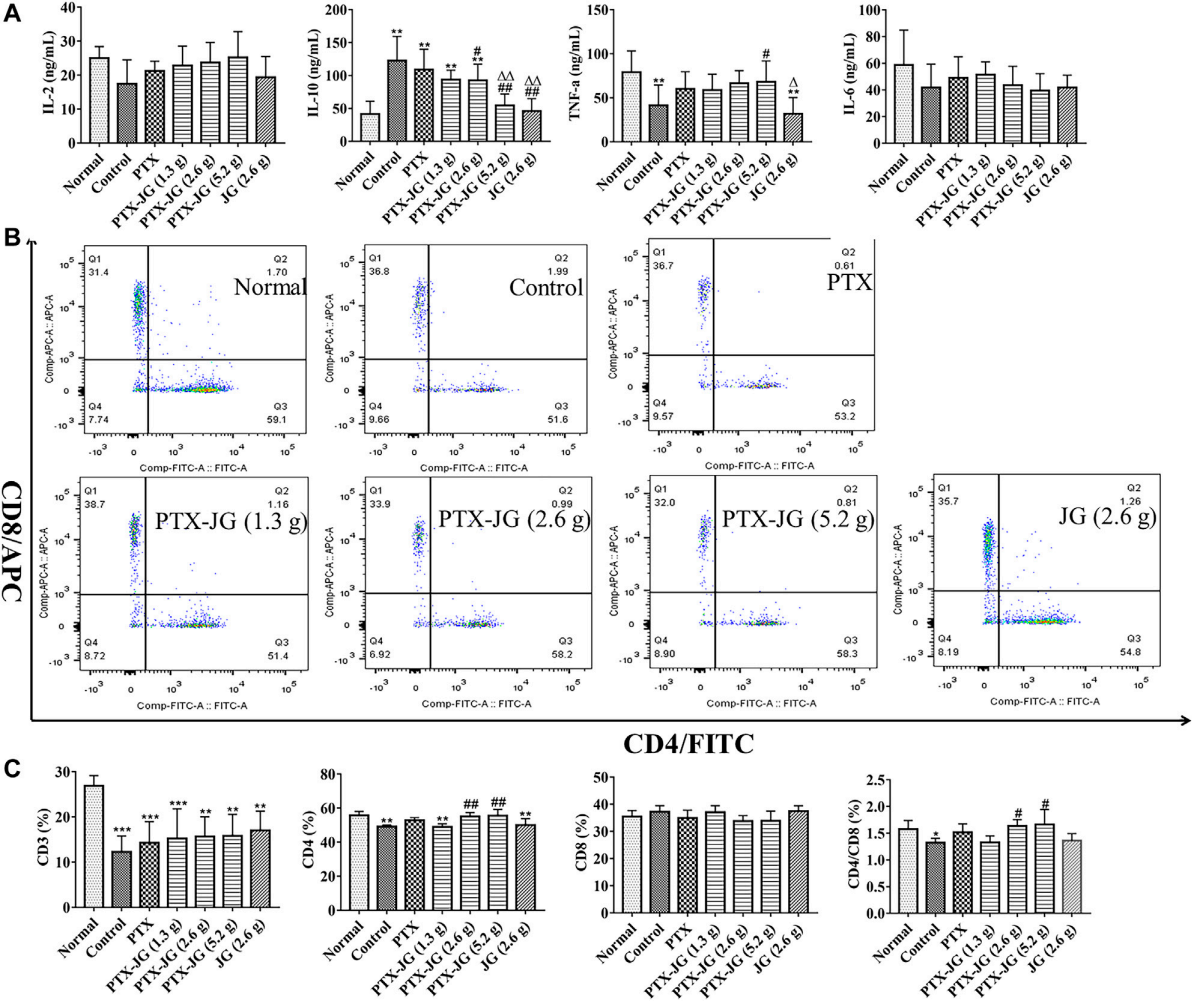
Immune indices of the combination of PTX and JG. **(A)**: IL-2, IL-10, IL-6 and TNF-α levels were detected by ELISA kit (n = 10). **(B)**: CD3^+^, CD4^+^ and CD8^+^ levels were detected by flow cytometry (n = 6). **(C)**: The ratios of CD3^+^, CD4^+^, CD8^+^ T cells and CD4+/CD8+ in spleen (n = 6). *p < 0.05, **p < 0.01, ***p < 0.001, *vs* Normal group; ^#^ *p* < 0.05, ^##^ *p* < 0.01, *vs* Control group; ^△^ *p* < 0.05, ^△△^ *p* < 0.01, *vs* PTX group.

To investigate the effect of the combination of JG and PTX on immunity, the immune cytokine levels in plasma were determined. IL-2 levels of mice in the tumor-bearing group were relatively low compared with those of mice in the normal group; however, this difference was not significantly different. Administration of the PTX-JG combination led to an increase in IL-2 levels compared with that in the PTX or control groups, although this change was not significant. IL-10 levels of mice in the control group were significantly higher (*p* < 0.01) than those in the normal group. IL-10 levels decreased after treatment with PTX. After administration of the combination of JG and PTX, IL-10 levels decreased significantly compared with those in the control and PTX groups (*p* < 0.01). TNF-α levels in mice in the tumor-bearing group were lower than those of mice in the normal group. TNF-α levels increased after the administration of PTX, but there were no significant differences between the PTX and control groups. TNF-α levels increased after PTX-JG intervention, especially after the administration of PTX-JG (5.2 g) when compared with the control group (*p* < 0.05). However, administration of JG (2.6 g) alone showed no effect on TNF-α levels. No significant differences among groups were observed with respect to IL-6 levels. The detailed results were showed in Figure 5A.

As shown in Figure 5B and Figure 5C, our results showed that CD3^+^ T cells in tumor-bearing mice were significantly decreased compared with those in normal mice (*p* < 0.001), indicating immune state deterioration in the pathological state. After PTX treatment, no aggravation of immune imbalance was observed in tumor-bearing mice. Treatment with JG alone did not show an obvious immunity enhancement in tumor-bearing mice; however, the ratios of CD4^+^ and CD4+/CD8+ in the spleen improved significantly after administration of the PTX-JG combination when compared with the control group (*p* < 0.01, *p* < 0.05). This finding further proved the synergistic effect of JG and PTX in restoring immune homeostasis.

### Biodistribution of Paclitaxel in Tumor Tissue

**FIGURE 6:**
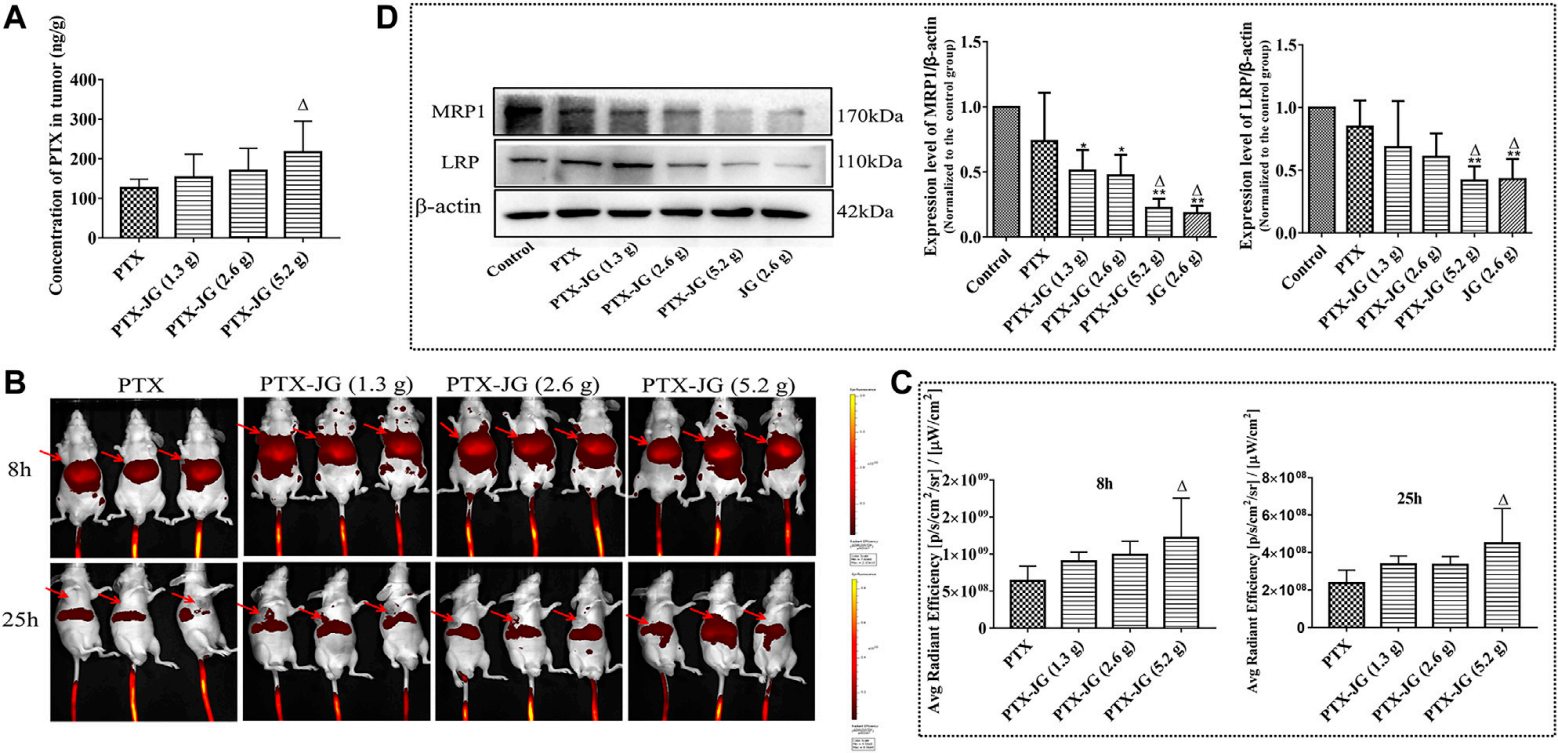
Effect of the combination of JG on the distribution of PTX in tumor. **(A)**: Content of PTX in tumor detected by HPLC-MS (n = 6). **(B)**: Fluorescence intensity near the tumor at 8 and 25 h after the combination of cy5.5-PTX with JG were detected with *in vivo* imaging system (n = 3), and the red arrows represented the location of the tumor. **(C)**: Region-of-interest (ROI) fluorescence intensities in tumor at 8 and 25 h (n = 3). **(D)**: Protein expression MRP1 and LRP were detected by Western blotting (n = 3). Values are expressed as the mean ± SD. ^#^ *p* < 0.05, ^##^ *p* < 0.01, vs Control group;^△^ *p* < 0.05, ^△△^ *p* < 0.01, *vs* PTX group.

As JG has meridian guide characteristics, we detected PTX levels in tumor tissues using LC-MS to determine if its enhanced antitumor effect was related to its increased distribution in tissues. The MS chromatograms of PTX in tumor tissue are shown in Supplementary Figure S2. As shown in Supplementary Figure S2, the method used to determine the specificity of PTX in tumor tissue met the requirements of biological sample testing, and no interfering substances were detected. The precision and recovery data of PTX in tumor were showed in Table 1. The linearity, repeatability, and stability data are presented in Supplementary Table S1, S2. As shown in Figure 6A, compared with the PTX group, PTX levels in the combination group increased with an increase in the JG dose, and PTX levels in the PTX-JG (5.2 g) group increased significantly (*p* < 0.05). *In vivo* imaging studies were performed to further confirm these phenomena. Luminescence intensities at the tumor sites were significantly increased in the combination group at 8 and 25 h compared with the sites in the PTX group. The detailed results were showed in Figure 6B and Figure 6C.

### Jiegeng Decoction Inhibited the Protein Levels of Lung Resistance-Related Protein and Multiresistance Protein 1 in Tumor Tissues

Biodistribution studies of PTX showed that when combined with JG, there was an increase in PTX levels in tumor tissue. Therefore, we speculated that the increase in PTX levels in tumor tissues was related to the JG-induced regulation of the drug-resistant protein in the tumor microenvironment. As shown in Figure 6D, the resistance proteins LRP and MRP1 in the control and PTX groups were upregulated and were significantly decreased (*p* < 0.01) in the JG group. LRP and MRP1 levels were significantly decreased (*p* < 0.01) when PTX was co-administered with JG.

### Effects of the Combination of Jiegeng Decoction and Paclitaxel Based on a Drug-Resistant Cell Model

#### Reversal Effect of Jiegeng Decoction on the Drug Resistance in A549/Paclitaxel Cells

**FIGURE 7:**
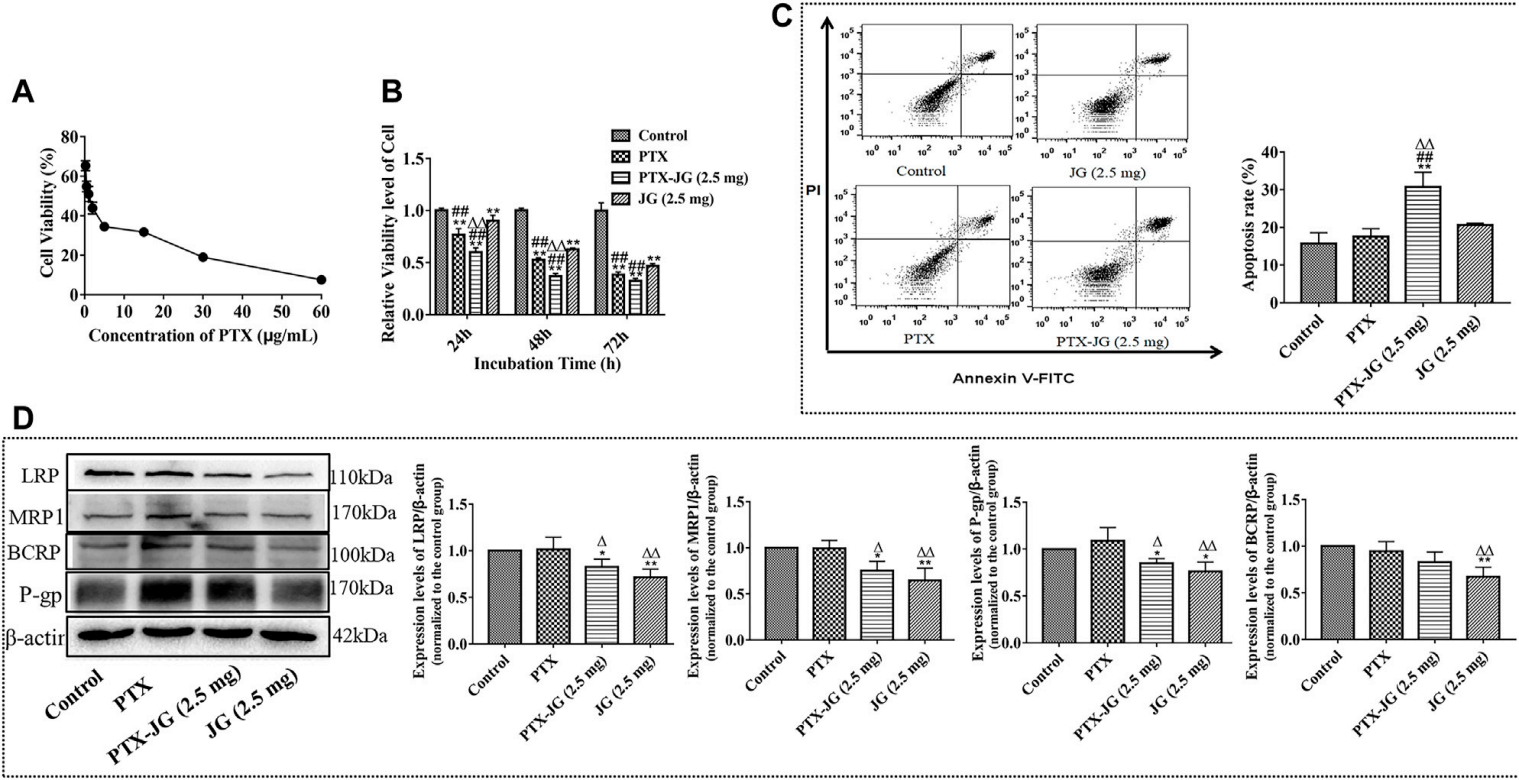
Effects of the combination of JG and PTX on A549/PTX tumor cells. **(A)**: Cell viability of A549/PTX tumor cells after the combination of JG (2.5 mg/ml) with PTX (60, 30, 15, 5, 2, 1, 0.5, 0.25 μg/ml) (n = 6). **(B)**: Effect of the combination of JG with PTX on the proliferation of A549/PTX tumor cells at 24, 48 and 72 h (n = 5). **(C)**: Effects of the combination of JG with PTX on apoptosis of A549/PTX tumor cells using Annexin V-FITC and PI staining (n = 4). **(D)**: Protein expression of LRP, MRP1, P-gp and BCRP of A549/PTX tumor cells were detected by Western blotting (n = 3). Values are expressed as the mean ± SD. *p < 0.05, **p < 0.01, *vs* Control group;^#^ *p* < 0.05, ^##^ *p* < 0.01, *vs* JG group;△p < 0.05, ^△△^ *p* < 0.01, vs PTX group.

A549/PTX cells were incubated in 96-well plates at a density of 2×10^3^ cells/well. As shown in Supplementary Figure S3, JG could inhibit the proliferation of A549/PTX cells in a dose-dependent manner. The inhibition rate of JG at the dosage of 2.5 mg/ml was less than 10%. Thus, 2.5 mg/ml was regarded as a noncytotoxic dose and selected as the dose for combination. Next, the effect of the combination of JG (2.5 mg/ml) and different concentrations of PTX (60, 30, 15, 5, 2, 1, 0.5, and 0.25 μg/ml) on A549/PTX cells was investigated. Figure 7A shows that the IC_50_ of the JG- PTX combination was 1.07 μg/ml, whereas that of PTX alone was 2.83 μg/ml, indicating a 2.64-fold reversal of drug resistance after the inclusion of JG.

#### Effect of the Combination of Jiegeng Decoction and Paclitaxel on the Proliferation of A549/Paclitaxel Tumor Cells

To investigate whether the enhanced antitumor efficiency was related to the inhibition of proliferation, the effect of the combination of JG and PTX on A549/PTX cell proliferation was examined. Cells were randomly divided into the control group, PTX group (0.5 μg/ml), PTX-JG (2.5 mg) group (JG (2.5 mg/ml) + PTX (0.5 μg/ml)). As shown in Figure 7B and Table 4, compared with the control group, both PTX and the JG-PTX combination could inhibit A549/PTX cell proliferation at 24, 48, and 72 h. At 24 h, JG treatment resulted in an obvious inhibition of A549/PTX tumor cell proliferation compared with cells in the control group; however, tumor cells in the JG group showed 90% survival, which can be regarded as inactive. At this time, the survival rate of tumor cells in the PTX group was 76%, while that in the PTX-JG (2.5 mg) group was about 60%. There was a significant difference between the PTX and PTX-JG (2.5 mg) groups (*p* < 0.01). At 48 h, the survival rate of tumor cells in the PTX group was still 50%, whereas that in the PTX-JG (2.5 mg) group was only about 35%, indicating that the combination of JG and PTX could have a synergistic effect on inhibiting tumor cell proliferation compared with the use of PTX alone (*p* < 0.01). At 72 h, the survival rate of A549/PTX cells in the PTX and PTX-JG (2.5 mg) groups further declined but the difference was not significant. In general, the combination of JG and PTX significantly inhibited the proliferation of A549/PTX cells and had a synergistic effect in inhibiting A549/PTX cell proliferation.

**TABLE 4:** Effect of JG on the proliferation of A549/PTX tumor cells.

Group	24 h		48 h		72 h	
	OD	SR (Survival rate, %)	OD	SR (Survival rate, %)	OD	SR (Survival rate, %)
Control	0.424 ± 0.010	100.00 ± 2.338	0.768 ± 0.018	100.00 ± 2.308	0.687 ± 0.050	100.00 ± 7.265
JG	0.382 ± 0.023^**^	90.035 ± 5.400^**^	0.481 ± 0.010^**^	62.604 ± 1.294^**^	0.322 ± 0.015^**^	46.899 ± 2.210^**^
PTX	0.325 ± 0.025^**##^	76.683 ± 5.874^**##^	0.404 ± 0.015^**##^	52.568 ± 1.910^**##^	0.264 ± 0.018^**##^	38.356 ± 2.587^**##^
PTX–JG (2.5 mg)	0.255 ± 0.017^**##△△^	60.136 ± 4.079^**##△△^	0.284 ± 0.019^**##△△^	36.951 ± 2.504^**##△△^	0.223 ± 0.016^**##^	32.418 ± 2.311^**##^

Data are presented as mean ± SD.

^*^ *p* < 0.05, ^**^ *p* < 0.01, vs. Control group; ^#^ *p* < 0.05, ^##^ *p* < 0.01, vs. JG group; ^△^ *p* < 0.05, ^△△^ *p* < 0.01, vs. PTX alone.

#### Combination of Jiegeng Decoction and Paclitaxel Exerted Pro-apoptosis Effects in A549/Paclitaxel Tumor Cells

To investigate the pro-apoptosis effect of the combination of JG and PTX, an annexin V-FITC/PI assay and flow cytometry were performed to analyze A549/PTX tumor cells. The top right quadrant represents the late apoptotic cells, the bottom left quadrant represents the viable cell population, and the bottom right quadrant represents early apoptotic cells. As shown in Figure 7C, PTX had a weak pro-apoptosis effect on A549/PTX tumor cells compared with the control group due to MDR. The apoptosis rate was only 15–20% and the pro-apoptotic effect of JG was maintained at a level similar to that of PTX. However, the combination showed a significant pro-apoptotic effect, further verifying the potential synergistic effect of JG and PTX.

#### Jiegeng Decoction Inhibited the Expression of MDR-Related Proteins in A549/Paclitaxel Cells

To further determine the effect of JG on the regulation of the MDR-related protein, the expression of BCRP, LRP, MRP1, and P-gp in A549/PTX cells were investigated. As shown in Figure 7D, the expression of BCRP, LRP, MRP1, and P-gp in A549/PTX cells increased. We also found that the expression of these resistance proteins in PTX group also maintained a high level, indicating that PTX was very likely to be insensitive to A549/PTX cells, and even stimulated the further expression of drug-resistant proteins in A549/PTX cells, such as p-gp. However, the expression of BCRP, LRP, MRP1, and P-gp in A549/PTX cells were significantly downregulated by JG than with PTX (*p* < 0.01, *p* < 0.05). After the combination of JG and PTX, the expression of LRP, MRP1, and P-gp in the combination group was significantly downregulated (*p* < 0.05) compared with the control group. BCRP expression in A549/PTX cells was also downregulated in the combination groups; however, the difference was not significant.

## Discussion

Chemotherapy failure involves in MDR. The main characteristics of MDR were the overexpression of MDR-related proteins including P-gp, MRP1, BCRP, and LRP in tumor tissues. Many chemotherapeutic agents, including PTX, are common substrates of P-gp and MRPs (Dinic et al., 2015), and the overexpression of P-gp or MRPs in tumor cells can reduce drug accumulation in cells. Thus, combining P-gp inhibitors or competing substrates of MRPs may enhance the distribution of chemotherapeutic agents and sensitize the pharmacological effects of PTX (Shi et al., 2019; Feng et al., 2020; Liu et al., 2020). JG has been commonly used to treat cough and other lung diseases clinically and is also a meridian guide drug. Studies have shown that the synergistic mechanism of meridian guide drugs is related to the regulation of MDR-related proteins (Leng et al., 2011; Liang et al., 2016; Wu et al., 2016). Therefore, we hypothesized that the combination of JG with PTX could down-regulate MDR-related protein and enhance the anticancer effect of PTX. Accordingly, we first determined the effect of JG on the distribution of PTX and our findings suggested that JG could increase PTX distribution in the liver, lungs, and heart. To further verify whether the JG-PTX combination could improve PTX distribution in lesion sites and enhance the anti-lung cancer effect of PTX, we further investigated the efficacy of this combination using a Lewis lung cancer model, and the distribution of PTX in tumor cells using HPLC-MS and *in vivo* imaging studies. The findings confirmed our hypothesis that JG increased PTX distribution in tumor cells and improved the anticancer effect of PTX. However, the mechanism of JG in enhancing the anti-lung cancer effect and the distribution of PTX in tumor cells has not been reported. Previous literatures showed that the active constituents in JG, such as Glycyrrhizic acid, Platycodin D, were established as P-gp inhibitor or substrate for P-gp, MRPs, and BCRP (Takara et al., 2005; Wakamatsu et al., 2007; Chen et al., 2009; Yang et al., 2015; Kwon et al., 2017), and may increase the absorption of the chemotherapy drug doxorubicin by inhibiting P-gp (Xu et al., 2021). Therefore, in this study, using a mouse model of Lewis lung cancer and an A549/PTX cell model, we further explored the effect of JG on MDR proteins, including P-gp, LRP, MRP1, and BCRP. Our findings found that JG alone could significantly decrease the expression of these efflux transporters, which suggested that JG, as a meridian guide drug, has a regulatory effect on drug-resistant proteins and is consistent with published findings of meridian guide drugs. Efflux transporters in tumor tissues and drug-resistant cells were down-regulated after the combination of JG and PTX, which could be the main causes for the improved distribution and increased efficacy of PTX. Thus, our results at least proved the feasibility of the combination of JG and PTX in overcoming the PTX resistance.

The efficacy of chemotherapy is also closely related to the body’s immunity. It is an indisputable fact that the immunity of cancer patients declines and deteriorates further after chemotherapy. Several studies have reported that Chinese medicines can enhance immunity. Jiegeng and Gancao and their active components activate macrophages and enhance immune function (Cheng et al., 2008; Zheng et al., 2017). Platycodon D triggers the release of extracellular PD-L1 and activates Jurkat T cells (Huang et al., 2019). Therefore, we investigated the effect of the combination of PTX and JG on immunity and found that it could significantly increase RBC, HGB, and PLT counts; increase IL-2 and TNF-α levels; and reduce IL-10 levels. CD4^+^ T cells can directly secrete large amounts of cytokines that kill tumor cells and inhibit tumor cell growth and proliferation. CD4^+^ T cells also play an auxiliary role in the stimulation and proliferation of CD8^+^ T cells and help CD8^+^ T cells generate long-term immunologic memory. To further clarify the mechanism of the combination on immune enhancement, the proportion of CD3^+^, CD4^+^, and CD8^+^ in spleen T cells was analyzed. In this study, we found that the PTX-JG combination improved the level of CD3^+^ and CD4^+^ T cells and maintained the CD4+/CD8+ immune balance despite the unobvious immune-enhancement effect of using JG alone. According to the data of secretion of cytokines, CD4^+^ T cells and CD4+/CD8+ ratio in the experiment, we speculate that the improvement of body immunity may also be one of the main causes for the improvement of anticancer effect of the PTX-JG combination group (Chen and Musa, 2021; Gao et al., 2021).

Drug-induced liver injury (DILI) is a major cause of acute liver failure and even death (Lee, 2003). It has been reported that PTX use can lead to impaired liver function (Vincenzi et al., 2018). Tissue-distribution experiments confirmed that JG could increase PTX distribution in the lungs, liver, and heart. Whether the increased distribution of PTX when administered in combination with JG leads to an increase in PTX toxicity in the corresponding organs has been the focus of research. Therefore, we further investigated the effects of the JG-PTX combination on liver function. Our findings suggested that the tumors themselves could cause abnormal liver function, and that liver function could be further damaged after chemotherapy. However, ALT and AST levels were downregulated after administration of the JG-PTX combination. Moreover, the use of this combination did not aggravate the potential PTX-induced liver injury despite its increased distribution in tissues. One of the reasons behind this phenomenon could be attributed to the detoxifying constituents of JG. Platycodon D, as the active component of Jiegeng, inhibits MAPK and apoptosis signaling pathways to reduce acetaminophen-induced acute liver injury (Fu et al., 2018). Licorice and its active components, diammonium glycyrrhizinate and magnesium isoglycyrrhizinate, improve DILI and enhance liver function (Yang et al., 2016; Luis et al., 2018; Zou et al., 2018). The myocardial injury index, CK-MB, was also determined and the findings suggested that the combination of JG with PTX did not affect cardiac function.

## Conclusions

In conclusion, the findings from this study confirmed the synergistic anticancer effect when JG was co-administered with PTX. We also found that the meridian guide effect of JG was related to the regulation of the drug-resistant protein. Thus, it is worth further exploring the combination of JG with other chemotherapy drugs for the treatment of liver and lung cancer, and to reduce the dosage of chemotherapy drugs by co-administering them with JG in an attempt to reduce side effects.

## Data Availability

The original contributions presented in the study are included in the article/Supplementary Material, further inquiries can be directed to the corresponding authors.
