# Supplementary material for: Jiegeng Decoction Potentiates the Anticancer Efficacy of Paclitaxel in vivo and in vitro
Source: Front Pharmacol. 2022 Feb 25;13:827520. doi: 10.3389/fphar.2022.827520 (PMC8914467; doi:10.3389/fphar.2022.827520)
Supplement: Supplementary file 1 [file Table1.docx]

| Supplementary Table 1 Regression data and LLOQ of the PTX in all matrices | | | | | |
| --- | --- | --- | --- | --- | --- |
|  | Linear regression equation | R | Linear range (ng/mL) | LLOQ (ng/mL) | LOD (ng/mL) |
| Heart | *y*=0.0000886*x* + 0.000134 | r=0.9991 | 20–4000 | 0.25 | 0.083 |
| Liver | *y*=0.0000802*x* + 0.000225 | r=0.9997 | 100–16000 | 0.5 | 0.125 |
| Spleen | *y*=0.00011*x* + 0.0000680 | r=0.9975 | 20–4000 | 0.25 | 0.083 |
| Lung | *y*=0.000047*x* + 0.00263 | r=0.9933 | 50–8000 | 0.25 | 0.083 |
| Kidney | *y*=0.0000708*x* + 0.000778 | r=0.9970 | 50–8000 | 0.25 | 0.083 |
| Tumor | *y*=0.00466*x* + 0.0432 | r=0.9962 | 25–4000 | 1 | 0.25 |

| Supplementary Table 2 Stability of PTX in tissue under various storage conditions (n =5) | | | | | | | | | | |
| --- | --- | --- | --- | --- | --- | --- | --- | --- | --- | --- |
| Samples | Concentration  (ng/mL) | Room temperature for 8 h | |  | Three freeze-thaw cycles | |  | | -20℃ for 30 days | |
|  |  | RSD (%) | RE (%) |  | 8.33 | 11.47 |  | RSD (%) | | RE (%) |
| Heart | 50 | 2.84 | -4.83 |  | 5.47 | 0.97 |  | 8.16 | | 14.45 |
|  | 500 | 8.28 | -1.37 |  | 5.16 | -3.78 |  | 5.76 | | -10.25 |
|  | 4000 | 10.71 | -0.09 |  | 2.85 | -1.77 |  | 3.74 | | -13.11 |
|  | 200 | 7.01 | -12.34 |  | 10.90 | -1.72 |  | 11.25 | | 11.45 |
| Liver | 2000 | 6.30 | 0.34 |  | 7.12 | 2.05 |  | 13.58 | | -0.61 |
|  | 16000 | 4.71 | 5.91 |  | 6.85 | 3.86 |  | 2.20 | | -14.11 |
|  | 50 | 3.98 | 8.39 |  | 5.11 | -4.82 |  | 3.41 | | 10.53 |
| Spleen | 500 | 7.25 | 14.19 |  | 4.17 | -4.78 |  | 5.81 | | -1.09 |
|  | 4000 | 4.65 | -3.00 |  | 4.89 | -5.87 |  | 3.49 | | -9.98 |
|  | 100 | 12.33 | -0.19 |  | 4.15 | -2.75 |  | 8.22 | | 5.02 |
| Lung | 1000 | 14.40 | 12.35 |  | 3.83 | 12.00 |  | 3.79 | | 4.56 |
|  | 8000 | 14.06 | 13.29 |  | 8.22 | -2.62 |  | 3.37 | | -3.47 |
|  | 100 | 6.21 | -4.25 |  | 3.41 | -1.96 |  | 6.59 | | 3.52 |
| Kidney | 1000 | 11.94 | -0.05 |  | 5.55 | 10.02 |  | 3.23 | | 2.22 |
|  | 8000 | 5.53 | 4.95 |  | 3.30 | 10.09 |  | 11.62 | | -10.25 |
| Tumor | 50 | 13.23 | -4.63 |  | 5.92 | 11.64 |  | 7.80 | | 0.38 |
|  | 200 | 7.04 | -7.51 |  | 2.88 | 7.09 |  | 7.25 | | 7.07 |
|  | 4000 | 11.10 | 0.77 |  | 9.35 | 3.72 |  | 5.10 | | 2.52 |


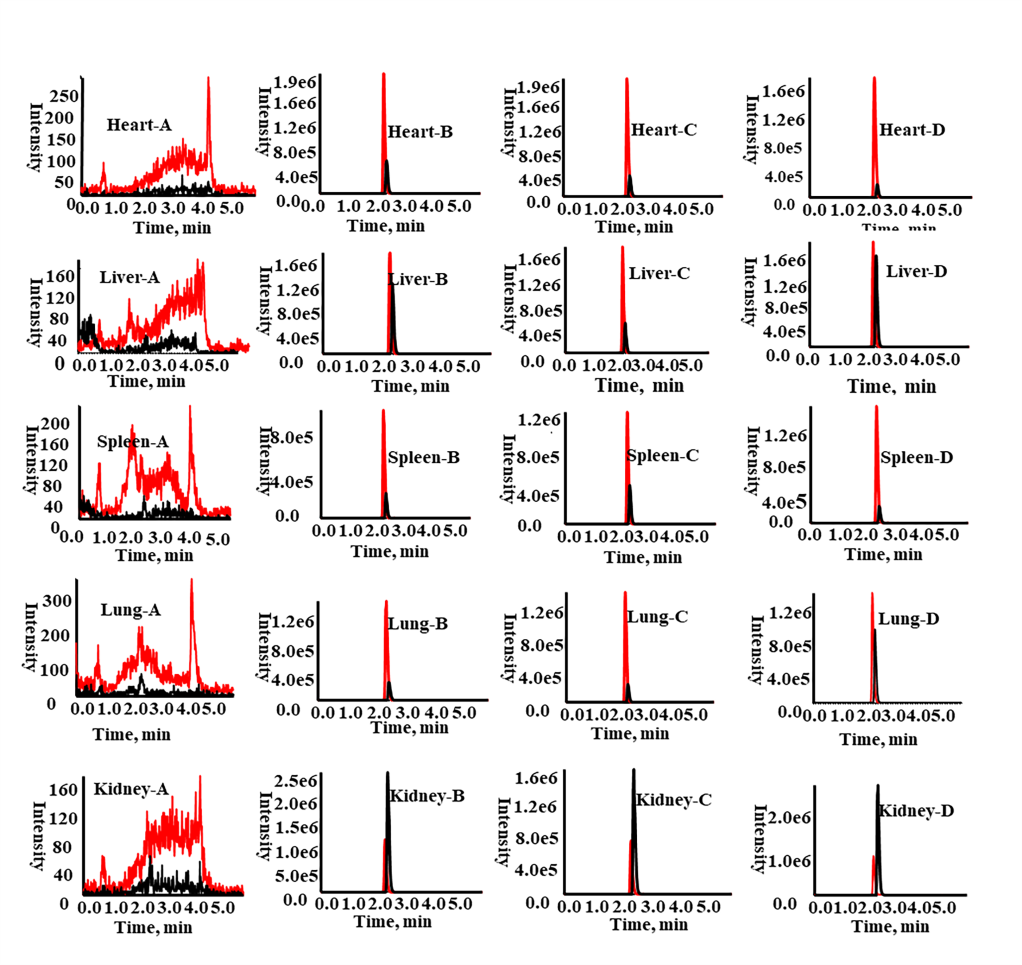


Supplementary Fig. 1 Specificity of the HPLC–MS method on the detection of PTX in tissues.

Blank tissue (A); Blank tissue spiked with PTX and ISs (B); Tissue sample after intravenous administration of PTX (C); Tissue sample after administration of PTX and JG (D).


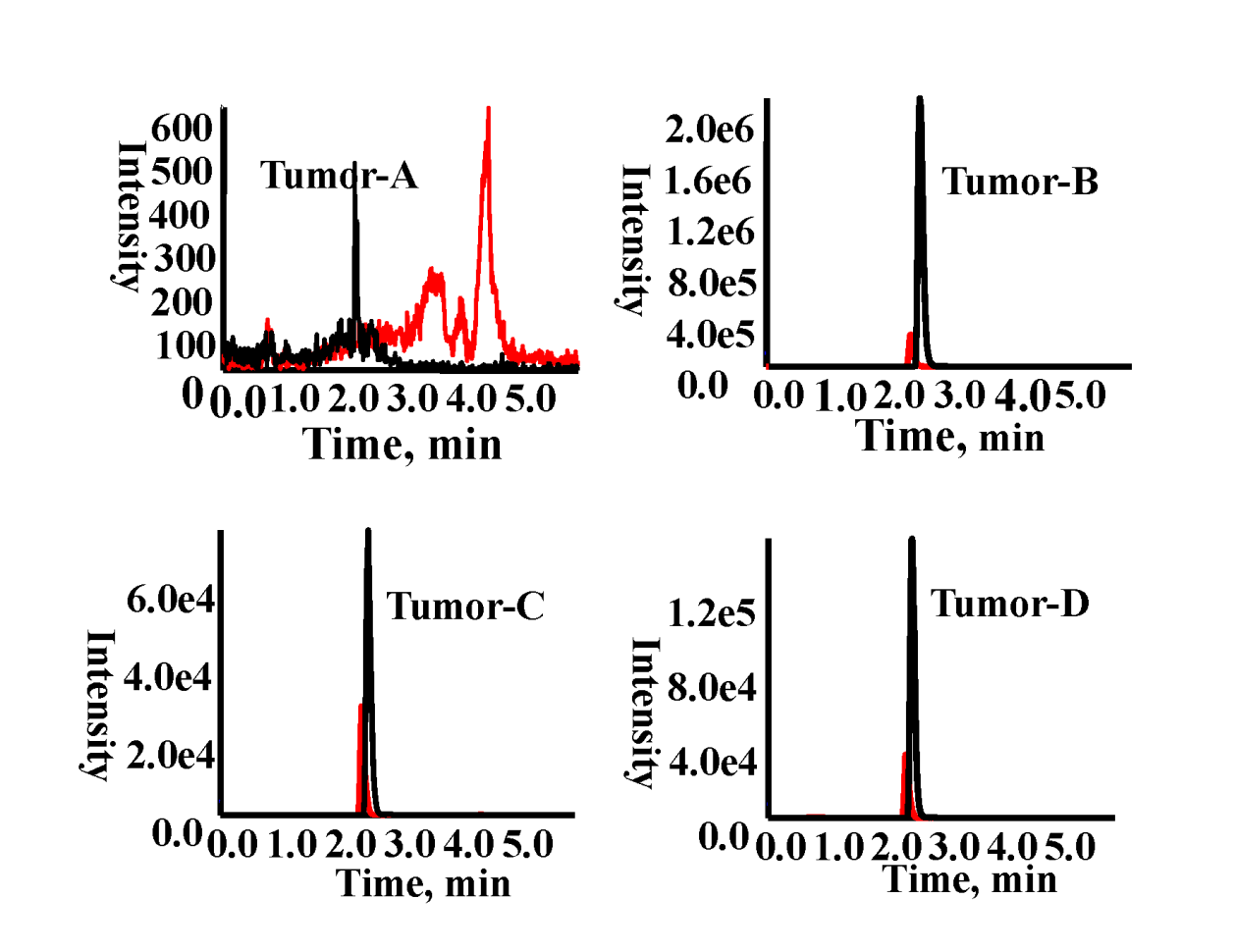


Supplementary Fig. 2 Specificity of PTX in tumor tissue using HPLC–MS

Blank tumor tissue (A); Blank tumor tissue spiked with PTX and ISs (B); Tumor tissue sample after intravenous administration of PTX (C);Tumor tissue sample after administration of PTX and JG (D).


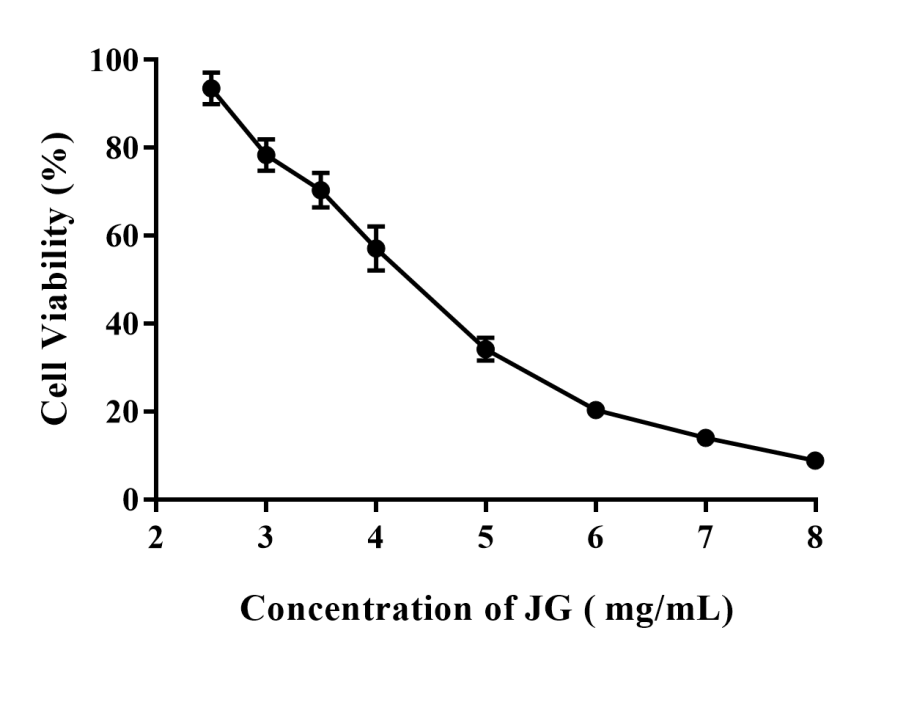


Supplementary Fig. 3 Effect of JG (8, 7, 6, 5, 4, 3.5, 3, 2.5 mg/mL) on the cell viability of A549/PTX tumor cell (n=6).
